# Immunization with recombinant fusion of LTB and linear epitope (40–62) of epsilon toxin elicits protective immune response against the epsilon toxin of *Clostridium perfringens* type D

**DOI:** 10.1186/s13568-019-0824-3

**Published:** 2019-07-12

**Authors:** Himani Kaushik, Sachin Kumar Deshmukh, Amit Kumar Solanki, Bharti Bhatia, Archana Tiwari, Lalit C. Garg

**Affiliations:** 1 Gene Regulation Laboratory, National Institute of Immunology, Aruna Asaf Ali Marg, New Delhi, 110067 India; 2 School of Biotechnology, Rajiv Gandhi Proudyogiki Vishwavidyalaya, Airport Bypass Road, Gandhi Nagar, Bhopal, 462036 India

**Keywords:** Sub-unit vaccine, *Clostridium perfringens*, Epsilon toxin, Heat labile enterotoxin, Neutralizing antibody

## Abstract

Epsilon toxin (Etx) produced by *Clostridium perfringens* types B and D, a major causative agent of enterotoxaemia causes significant economic losses to animal industry. Conventional vaccines against these pathogens generally employ formalin-inactivated culture supernatants. However, immunization with the culture supernatant and full length toxin subjects the animal to antigenic load and often have adverse effect due to incomplete inactivation of the toxins. In the present study, an epitope-based vaccine against *Clostridium perfringens* Etx, comprising 40–62 amino acid residues of the toxin in translational fusion with heat labile enterotoxin B subunit (LTB) of *E. coli*, was evaluated for its protective potential. The ability of the fusion protein rLTB.Etx_40–62_ to form pentamers and biologically active holotoxin with LTA of *E. coli* indicated that the LTB present in the fusion protein retained its biological activity. Antigenicity of both the components in the fusion protein was retained as anti-fusion protein antisera detected both the wild type Etx and LTB in Western blot analysis. Immunization of BALB/c mice with the fusion protein resulted in a significant increase in all isotypes, predominantly IgG1, IgG2a and IgG2b. Anti-fusion protein antisera neutralized the cytotoxicity of epsilon toxin both in vitro and in vivo. Thus, the results demonstrate the potential of rLTB.Etx_40–62_ as a candidate vaccine against *C. perfringens*.

**Electronic supplementary material:**

The online version of this article (10.1186/s13568-019-0824-3) contains supplementary material, which is available to authorized users.

## Introduction

*Clostridium perfringens,* the causative agent of enteric diseases in domestic animals, is a non-motile, spore-forming, Gram-positive, anaerobic bacterium (Songer 1996; Smedley et al. 2004). Based on different types of toxin produced by the strains, *C. perfringens* was classified into 5 groups (A to E) (Uzal et al. 2014). However, recently it has been proposed to classify the organism into 7 types (A–G) (Rood et al. 2018). *C. perfringens* types B and D produce epsilon toxin that generally affects animals such as sheep, goats, and cattle. Type D can also cause enterocolitis in adult goats as well as enterotoxaemia in calves (Stiles et al. 2013). Epsilon toxin is one of the most toxic clostridial toxins after botulinum and tetanus toxins. *C. perfringens* types B and D produce a large amount of epsilon toxin in the gut of infected animals which upon absorption by the gut mucosa results in elevated blood pressure and severe vascular damage due to increased vascular permeability as well as lesions in various organs predominantly the brain, heart, lung and kidney (Petit et al. 2003; Tamai et al. 2003; Miyamoto et al. 2008; Popoff 2011).

Due to the high toxicity of the toxin and rapid progression of the disease, the animals die shortly after appearance of symptoms making antibiotic treatment ineffective. Therefore, to protect the animals from the fatal effects of the toxin, vaccination remains the most effective method of control. It is well established that the epsilon toxin is the sole causative agent and an absolute requirement for the *C. perfringens* to cause the disease in sheep and goats (Garcia et al. 2013). It has been shown that the epsilon toxin (Etx) null mutant strains of *C. perfringens* type D failed to cause the disease and complementation with wild type Etx restored their virulence (Garcia et al. 2013). Thus, though the other toxins produced by *C. perfringens* type D were present, mutation in Etx resulted in failure of the organism to induce the disease. Further, it has been shown that immunization with formaldehyde inactivated recombinant Etx adjuvanted with 0.05% aluminum hydroxide gel (algel) conferred protection against the *C. perfringens* infection (Chandran et al. 2010). Thus, the Etx plays a key role in *C. perfringens* virulence and is necessary for the organism to induce disease. Currently employed vaccines against *C. perfringens* epsilon toxin include toxoid based vaccine using formalin-inactivated culture supernatants and inactivated whole cell lysate (Chandran et al. 2010). Immunization with the toxoid results in an antigenic load, leading to a non-specific immune response, thus making the immune response sub-optimal thereby incapable of evoking desired protection against the infection. Immunization with purified recombinant epsilon toxin and its mutants has been evaluated for their vaccine potential (Bokori-Brown et al. 2014). However, this often generates a local inflammatory reaction and the dose of the toxin has to be monitored carefully so as not to cause adverse effects. Use of small segments of the antigen comprising immunodominant regions as a vaccine candidate would possibly help overcome this problem. Owing to their small size, the epitopes are not immunogenic by themselves, however, they are able to generate focused immune responses against the targeted antigens when coupled with the suitable carrier. Such epitope-based vaccines conjugated to carrier molecules have been successfully used against a variety of pathogens including viruses, bacteria and parasites (Stoloff and Caparros-Wanderley 2007; Skwarczynski et al. 2012; Cong et al. 2014; Sharma and Dixit 2016). The B subunit of heat labile enterotoxin (LTB) of *Escherichia coli* is highly immunogenic and self adjuvanting (Weltzin et al. 2000; Sharma and Dixit 2015). LTB has been reported to enhance the immune responses of non-immunogenic antigens when used as a carrier. Co-administration of LTB or translational fusion of LTB with the targeted antigen has been reported to augment immune responses (Qiao et al. 2009; Todoroff et al. 2013). Fusion of immunodominant epitopes of human papillomavirus-16 L1 antigen with LTB retained GM1 ganglioside receptor binding and elicited high immune response (Waheed et al. 2011). Oral and parenteral immunization with a fusion protein comprising two immunodominant epitopes of hepatitis B virus surface antigen and LTB resulted in high serum IgG response against both the fusion partners (Schodel et al. 1991). The reports by these workers showed that fusion with LTB resulted in improved immunogenicity and thus have potential to be used as a low cost adjuvant in vaccine development.

We have earlier reported cloning of one of the immunodominant epitopes (spanning 40–62 amino acid residues) of epsilon toxin of *C. perfringens*, in translational fusion with LTB for its secretory expression (Kaushik et al. 2013). The present study was conducted to optimize production and purification of the purified fusion protein in large amounts from the culture supernatants of *V. cholerae* cells and to evaluate the vaccine potential of the fusion protein against *C. perfringens* epsilon toxin.

## Materials and method

### Expression and purification of secretory fusion protein rLTB.Etx_40–62_

*Vibrio cholerae* JBK70 cells (ATCC Number: 39318) were a kind gift from Dr. M. Lewin, University of Maryland School of Medicine, Baltimore, USA. The *V. cholerae* JBK70 cells harbouring the recombinant plasmid pMMBltbEtx_40–62_ were induced with IPTG essentially as described earlier (Kaushik et al. 2013) and the recombinant fusion protein was purified from the induced culture supernatants as described earlier (Mekalanos et al. 1978). To facilitate purification of the fusion protein, other proteins were removed by 30% ammonium sulphate precipitation. The fusion protein present in the supernatant was then collected by precipitation with 70% ammonium sulphate followed by centrifugation at 12,000×*g* at 4 °C for 40 min. The pellet was resuspended in 10 mM sodium phosphate buffer, pH 7.4 and dialysed extensively against the same. The dialyzed protein was then subjected to cation-exchange chromatography using cellulose phosphate cation exchanger resin (Sigma Aldrich Chemical Co., USA). Proteins were eluted with sodium phosphate buffer of different strengths (100 mM, 200 mM, 300 mM and 400 mM), pH 7.4. Different fractions were analyzed on SDS-PAGE (15%) at constant current of 30 mA at room temperature (25 °C), and the fractions showing the presence of the fusion protein were pooled and dialyzed against 10 mM phosphate buffer, pH 7.4. The purified protein was flash frozen and stored at − 20 °C until further use.

### Purification of recombinant Etx

The method described by Mathur et al. (2010) was followed for the purification of recombinant Etx (rEtx). Briefly, the *E. coli* M15 cells (Qiagen, USA) transformed with pQE60etx plasmid were grown till the absorbance of the culture at 600 nm reached 0.6. The cells were then induced with 1 mM IPTG (isopropyl-β-d-thiogalactoside) and grown further for 6 h. The induced culture was then subjected to centrifugation at 3000×*g* for 5 min at 4 °C, the supernatant was discarded and the cell pellet resuspended in 10 mM Tris–HCl, pH 7.4 was sonicated for 10 cycles each of 30 s each with cooling for 30 s between the cycles, in a sonicator (Misonix, USA). The cell lysates thus obtained were then centrifuged (12,000×*g* for 15 min at 4 °C) to obtain the soluble fraction and insoluble fraction. Anion exchange chromatography using diethylaminoethyl (DEAE)-Sepharose (Amersham Pharmacia, USA) was used to purify the rEtx from the soluble fraction. The column was pre-equilibrated with 10 mM Tris–HCl, pH 8.0 prior to loading of the soluble fraction. The sample was then loaded onto the preequilibrated column followed by thorough washing (4 column volumes) with 10 mM Tris–HCl, pH 8.0. Bound proteins were eluted using a continuous pH gradient of 10 mM of Tris–HCl in the pH range between pH 8.0 to pH 5.0). Different fractions were analyzed by SDS-PAGE (12%) and the fractions showing the presence of rEtx were pooled. The protein concentration was determined using the BCA protein estimation kit (Pierce, USA).

### Purification of recombinant LTB (rLTB)

For the purification of rLTB, the *V. cholerae* cells harboring the recombinant plasmid pMBLTB [available in the lab, Kaushik et al. (2013)] were induced with 1 mM IPTG (isopropyl-beta-d-thio-galactopyranoside) for 6 h and the rLTB was purified from the culture supernatant by the method of Mekalanos et al. (1978). Briefly, 2 l of the induced culture cells were harvested at 6000×*g* for 15 min at 4 °C. The supernatant was collected and the non-specific proteins were salted out with 30% ammonium sulphate and 70% ammonium sulphate precipitation. The pellet obtained after 70% saturation was solubilized in 10 ml of 10 mM sodium phosphate buffer (pH 7.4) and loaded onto cellulose phosphate column [equilibrated with the 10 mM sodium phosphate buffer (pH 7.4)]. The bound proteins were eluted using 200 mM sodium phosphate buffer, pH 7.4 (30 fractions of 1 ml each). The collected fractions were analysed on 15% SDS-PAGE to check the purified protein and dialysed against the 10 mM phosphate buffer (pH 7.4).

### Circular dichroism (CD) spectroscopy

The solution conformation of the purified fusion protein rLTB.Etx_40–62_ was investigated using CD spectroscopy according to (Mathur et al. 2010) with some modifications. CD Spectra of the fusion protein (0.2 mg/ml in 10 mM phosphate buffer, pH 7.4) were recorded at a scan speed of 50 nm/min in JASCO-J815 spectropolarimeter fitted with a temperature controller (PTC-348 W) at 25 °C using a quartz cuvette of 0.1 cm pathlength in far UV-region (200–250 nm). At least 10 spectra were recorded for each sample (LTB alone and purified fusion protein) and averaged after baseline subtraction (buffer spectra). Mean residue weight ellipticities were calculated and expressed in units of degree cm^2^ dmol^−1^.

### Biological activity assay of the rLTB.Etx_40–62_

In vitro biological activity of the LTB component in the purified rLTB.Etx_40–62_ was evaluated using Chinese Hamster Ovary-K1 (CHO-K1, procured from the National Centre for Cell Science, Pune, India) cells as described earlier (Takeda et al. 1981). Prior to biological activity assay, the recombinant fusion protein (rLTB.Etx_40–62_) and wild type LTB were mixed with recombinant heat labile enterotoxin A subunit (LTA) to constitute the holotoxin. CHO-K1 cells were cultured in DMEM supplemented with 1% FCS, 2 mM glutamine, penicillin (100 U/ml) and streptomycin (100 µg/ml) at 37 °C in 5% CO_2_ humidified atmosphere. The CHO-K1 cells (2 × 10^4^ cells/ml/well in a 24 well tissue culture plate) were treated with equal amounts of the reconstituted fusion protein holotoxin and wild type holotoxin and incubated for 16 h. Morphological examination of the cells was performed under a light microscope.

### Immunization of BALB/c mice with rLTB.Etx_40–62_

After collection of pre-immune sera, a group of BALB/c mice (n = 6; body weight = ~ 20 g) were immunized with the rLTB.Etx_40–62_ (1 μg/g body weight) emulsified in alum. Pre-immune sera were collected prior to immunization. Four weeks after primary immunization, two booster doses with the same amount of protein emulsified in alum were administered at 2 weeks intervals. Mice were bled 1 week after 2nd booster. The blood was allowed to clot for 1 h at room temperature (RT) and the sera were collected by centrifugation at 10,000×*g* for 10 min at RT and stored in small aliquots at − 20 °C.

### ELISA for antibody titer determination and antibody isotyping

To determine the antigenic specificity of the antisera, antigen specific ELISA was performed as described by Sharma and Dixit (2015). Briefly, purified recombinant epsilon toxin [rEtx, 500 ng/100 μl of coating buffer (0.2 M carbonate–bicarbonate buffer, pH 9.2)] was coated in 96 well microtiter plates and incubated for 1 h at 37 °C. Non-specific sites were blocked with 5% non-fat milk in 1× PBST [0.05% Tween-20 in 1× PBS (140 mM NaCl, 3 mM KCl, 10 mM Na_2_HPO_4_, 1.5 mM KH_2_PO_4,_ pH 7.4)] for 1 h at RT with shaking (100 rpm). Different dilutions of the sera in PBS with 2% BSA (100 μl/well) were added to each well and incubated for 2 h at 37 °C. This was followed by the addition of 100 μl of HRP-conjugated anti-mouse IgG (1:10,000 in 1× PBS containing 2% BSA) to each well and incubated at 37 °C for 1 h. Wells were washed thrice with 1× PBST (10 min each) between successive incubations. Color was developed by addition of 100 μl orthophenylenediamine (OPD; 0.5 mg/ml in citrate phosphate buffer, pH 5.5) supplemented with hydrogen peroxide (1 μl/ml). The reaction was terminated by the addition of 50 μl of 2 N H_2_SO_4_ and the absorbance was measured against PBS containing 0.2% BSA (optical blank) at 490 nm using BioTek microplate reader.

For antibody isotyping, titers for a particular isotype of IgG were checked in pooled sera. Briefly, a 96 well plate was coated with an excess of purified fusion protein and incubated with the pooled mouse sera, followed by incubation with biotinylated anti-mouse IgG_1_, IgG_2a_ or IgG_2b_ antibodies (1:1000). Subsequent incubation was done with streptavidin-HRP (1:1000) and developed using orthophenylenediamine (OPD) as described earlier (Kaushik et al. 2018). The reaction was terminated by adding H_2_SO_4_ and absorbance was measured at 490 nm.

### Immunoblotting

Immunoblotting was essentially performed as described by Towbin et al. (1979). Recombinant Etx was resolved on SDS-PAGE (12%) and electro-transferred onto nitrocellulose membrane at 30 mA for 10 h at 4 °C in transfer buffer (48 mM Tris, 39 mM glycine, 0.037% SDS, 20% methanol). Non-specific sites were blocked by incubating the membrane in blocking solution (1% non-fat milk in 1× PBST) for 1 h. The membrane was then incubated at RT for 2 h with primary antibody (1:1000) in blocking solution and washed three times with 1× PBST, followed by incubation with HRP-conjugated secondary antibody (1:2000) at RT for 1 h. The blot was thoroughly washed with 1× PBST between successive incubations. Colorimetric detection was done with diaminobenzidine (DAB) (0.5 mg/ml in 1× PBS) and the immunoreactive protein band was developed by the addition of hydrogen peroxide (1 μl/ml) and the reaction was terminated by washing the membrane with water.

### In vitro and in vivo neutralization analysis of anti-fusion protein antisera

The neutralizing ability of the fusion protein antisera (anti-rLTB.Etx_40–62_) against the Etx was determined essentially as described previously (Beal et al. 2003; Gil et al. 2015). Briefly, Madin-Darby Canine Kidney epithelial (MDCK, procured from the National Centre for Cell Sciences, Pune, India) cells were seeded (2 × 10^4^ cells/100 μl/well) in a 96 well cell culture plate in 10% DMEM [Dulbecco’s modified Eagle’s medium supplemented with 10% (v/v) heat-inactivated fetal bovine serum]. Different dilutions (neat, 1:10, 1:25, 1:50, 1:100, 1:200 and 1:500) of the anti-rLTB.Etx_40–62_ antisera collected after the 2nd booster were incubated with 7.5 ng of rEtx for 1 h at 37 °C prior to the addition to MDCK cells. The cells treated with the antisera-toxin mixture were maintained at 37 °C and 5% CO_2_ for 2 h in a humidified incubator. Cell viability was checked by staining the cells with metabolic indicator 3-(4,5-dimethyl-2-thiazolyl)-2,5-diphenyl-2*H*-tetrazolium bromide (MTT) (Sigma Aldrich Chemical Co., USA) as described by Mosmann (1983). The rEtx incubated with undiluted pre-immune sera (PI, neat) was included as a negative control. Percent survival was calculated with respect to untreated cells.

Neutralizing potential of the antisera was also assessed by challenging the mice with rEtx pre-incubated with different antisera namely pre-immune sera, anti-fusion protein antisera generated with or without alum as an adjuvant. Before the experiment, LD50 dose of the rEtx was determined by administering different groups of mice (n = 6 each group) with different concentrations of the rEtx [0.5 ng, 1 ng, 1.5 ng, 2 ng, 2.5 ng, 3 ng, 3.5 ng and 4 ng (in 5 µl PBS) per 20 g body weight] and mice were monitored for survival. The LD50 dose of the rEtx was thus determined to be 1.5 ng/20 g body weight. To assess the neutralization potential of the antisera in mice, pre-determined 2 × LD50 dose of the rEtx (3 ng/20 g body weight in 5 µl PBS) was pre-incubated with equal volume of the undiluted neat antisera for 30 min at 37 °C and administered (intraperitoneally) into BALB/c mice (n = 10 per group). Mice administered with the rEtx preincubated with equal volume of preimmune serum were included as control. The mice were monitored for survival for 5 days.

### Statistical analysis

Statistical analysis to determine the significance values (*p* value) was performed by employing one way or two-way analysis of variance (ANOVA) test (Dunnett’s multiple comparison test) using GraphPad Prism 7 software (Chicago, IL, USA). The data show mean ± SD of three independent experiments performed in triplicates.

### Gene sequence accession numbers

Nucleotide sequences of the Etx, LTA and LTB used in the present study have been submitted in NCBI by GenBank Accession no. AJ426474.1 and MF990203 and M17874.1, respectively. The plasmid pMMBltbEtx_40–62_ harbors the DNA region encoding the 40–62 amino acid residues of the Etx in translational fusion with the LTB cloned in PMMB68 plasmid, construction of which has been described earlier by Kaushik et al. (2013). Sequence of the fusion gene construct has been submitted to GenBank (Accession No. MH845624).

## Results

### Purification of secretory fusion protein

**Fig. 1:**
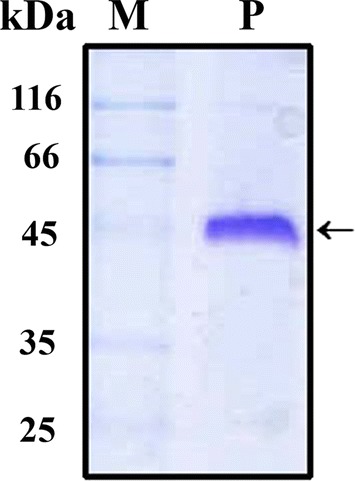
SDS-PAGE (12%) analysis of purified rLTB.Etx_40–62_. Lane P shows the purified rLTB.Etx_40–62_ resolved under non-denaturing conditions in the absence of beta mercaptoethanol. Lane M shows migration of protein molecular weight markers (kDa). Arrow points to the pentameric recombinant fusion protein

For evaluation of epitope based vaccine against Etx, large scale purification of the recombinant fusion protein rLTB.Etx_40–62_ was carried out from the culture supernatants of the induced *V. cholerae* JBK70 cells harbouring the pMMBltbEtx_40–62_ (Kaushik et al. 2013). Ammonium sulphate precipitation of the culture supernatant resulted in enrichment and partial purification of the rLTB.Etx_40–62_. Cation exchange chromatography of the ammonium sulphate (70%) precipitated proteins resulted in purification of the rLTB.Etx_40–62_ to near homogeneity (Fig. 1, lane 1). Approximately, 25 mg of purified soluble rLTB.Etx_40–62_ could be obtained from 1 l of culture at shake flask level.

### Secondary structure analysis of the fusion protein

**Fig. 2:**
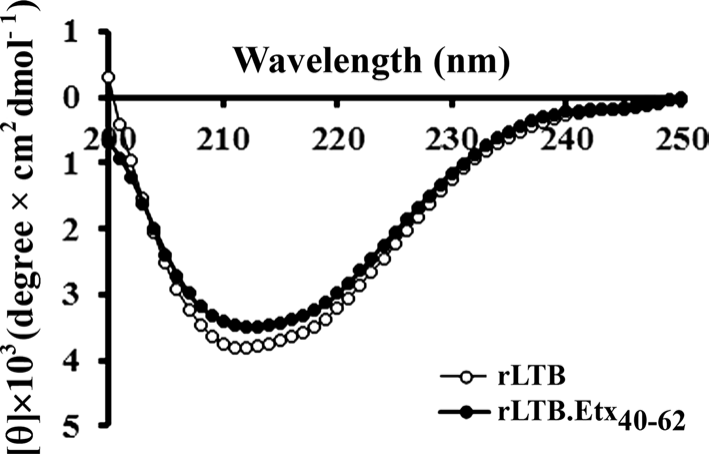
Secondary structure analysis of rLTB.Etx_40–62_: far UV circular dichroism spectra of the rLTB.Etx_40–62_ and rLTB recorded in the wavelength range 200–250 nm are shown. Mean residue mass ellipticity (θ) in units of degree × cm^2^ dmol^−1^ are depicted

To assess the effect of genetic fusion of epsilon toxin epitope at the C-terminal of LTB on its conformation, secondary structures of the fusion protein and the wild type LTB were analysed by CD spectroscopy (Greenfield 2006). As evident from Fig. 2, structural profile of the rLTB.Etx_40–62_ was similar to that of the wild type LTB. Secondary structure prediction using Reed’s method (Reed and Reed 1997) revealed the fusion protein to have 43.19 ± 3.22% β-sheet, 10.48 ± 3.74% β-turns, 44.72 ± 1.55% random coil, and 1.09 ± 0.103% α-helix. The secondary structure of the fusion protein was not found to be significantly different from that of the wild type rLTB which exhibited to have 42.77 ± 1.25% β-sheet, 8.75 ± 1.28% β-turns, 47.13 ± 0.18% random coil, and 1.36 ± 0.78% α-helix.

### Biological activity of in vitro assembled rLTA-rLTB.Etx_40–62_ holotoxin

**Fig. 3:**
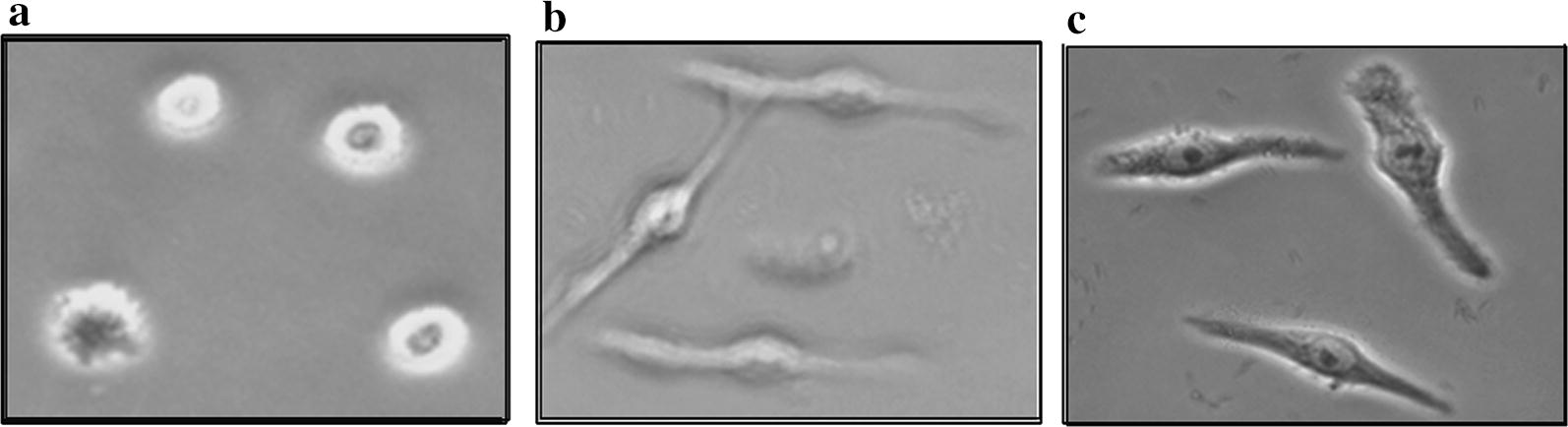
CHO-K1 cells elongation activity of in vitro assembled holotoxins: ability of the rLTB.Etx_40–62_ to form functional holotoxin was adjudged by incubating CHO-K1 cells with in vitro assembled holotoxins. CHO-K1 cells (1 × 10^4^ cells/well/100 µl) in a 96 well plate were treated with the reconstituted holotoxins (10 ng/ml) for 16 h at 37 °C in the presence of 1% fetal calf serum under 5% CO_2_ humidified conditions and visualized under light microscope (×100). **a** PBS-treated CHO-K1 cells (negative control). **b** In vitro assembled holotoxin comprising rLTA and wild type rLTB (positive control). **c** In vitro assembled holotoxin comprising rLTA and rLTB.Etx_40–62_ fusion protein

In order to assess if the rLTB.Etx_40–62_ formed a functional holotoxin with the wild type LTA, effect of the reconstituted holotoxin (rLTA + rLTB.Etx_40–62_, Fig. 3c) comprising the fusion protein was evaluated on the morphology of CHO-K1 cells and was compared with that of wild type holotoxin (rLTA + rLTB, Fig. 3b). As evident from the figures, both the holotoxins were able to elongate approximately 95% CHO-K1 cells at 10 ng concentration.

### Analysis of immune response

**Fig. 4:**
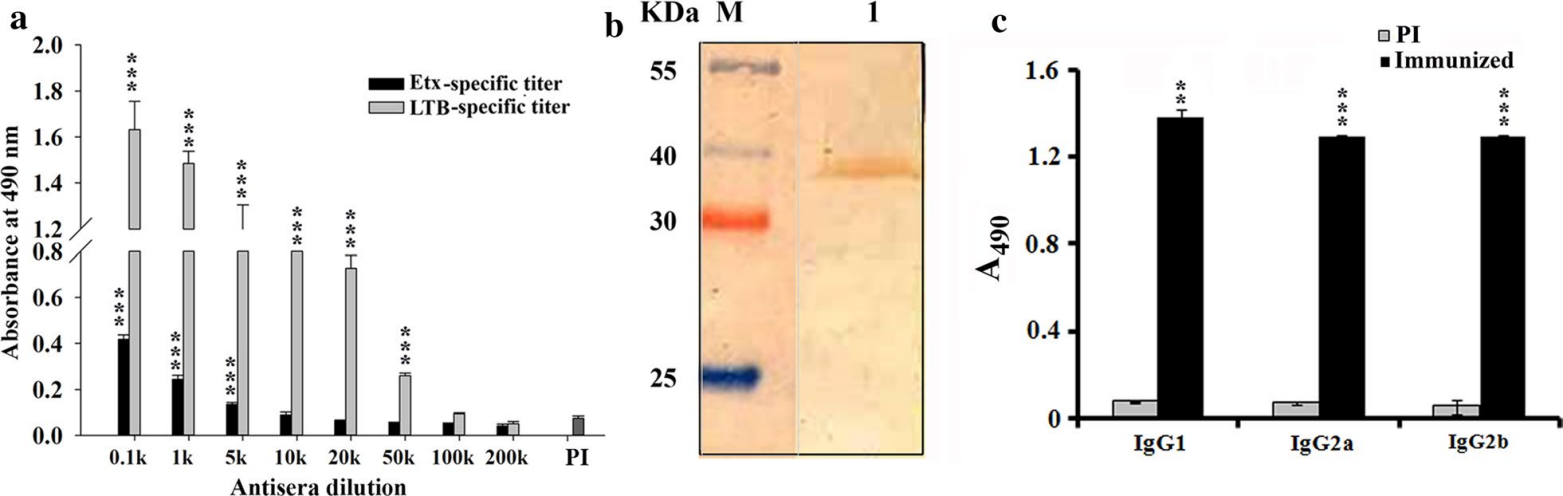
**a** Antibody titer of the anti-rLTB.Etx_40–62_ antisera against LTB and Etx. ELISA was carried out to determine the LTB-specific and Etx-specific antibody titers in the anti-fusion protein antisera by coating the respective proteins (500 ng/well) as target. Anti-fusion protein antisera (1:2000) was then added followed by the addition of secondary anti-mouse HRP-conjugated antibody. The color was developed by the addition of the substrate OPD and the absorbance was measured at 490 nm. Statistical difference (*p* value) was calculated using ordinary two-way ANOVA as compared to preimmune serum (PI). **b** Immunoblot analysis of the rEtx with the anti-rLTB.Etx_40–62_ antisera. Purified rEtx (lane 1) was immunoblotted with anti-rLTB.Etx_40–62_ (1:5000 in 1× PBS) antisera. Distinct immunoreactive band indicates that the antibodies present in the anti rLTB.Etx_40–62_ antisera are able to efficiently crossreact with rEtx. Lane M indicates migration of protein molecular weight (kDa). **c** Antigen-specific antibody isotyping of the anti-rLTB.Etx_40–62_ antisera. Different antibody isotypes in anti-rLTB.Etx_40–62_ antisera (1:10,000 in 1× PBS) collected on 28 days post immunization were analyzed by ELISA using biotinylated isotype specific anti-mouse IgG_1_, IgG_2a_ and IgG_2b_ (1:5000) secondary antibodies. Pre-immune (PI) sera collected prior to immunization was included as control. Statistical difference (*p* value) for each isotype in the anti-fusion protein antisera was calculated using Student’s two-tailed t-test as compared to that measured in preimmune serum (PI). **p ≤ 0.005; ***p ≤ 0.001

Immunization with the fusion protein rLTB.Etx_40–62_ comprising of an epitope of Etx and LTB emulsified in alum adjuvant, generated significant immune response against both the LTB and Etx. Antibody titer determination showed the fusion protein to be highly immunogenic as the antisera with very high end point titers of > 1:100,000 were obtained against the fusion protein. ELISA using the parent proteins Etx and LTB also showed significantly increased absorbance (*p* ≤ 0.001 with LTB from 0.1 × 10^3^ to 50 × 10^3^-fold dilutions and with rEtx from 0.1 × 10^3^ to 5 × 10^3^-fold dilutions) in comparison to the preimmune serum. The antigen specific end point titers in the fusion protein antisera were determined to be ~ 1:10,000 and > 1:100,000 for Etx and LTB, respectively (Fig. 4a).

ELISA results showed that the anti-rLTB.Etx_40–62_ antisera could also recognize the fusion protein (rLTB.Etx_40–62_) as well as the rEtx efficiently. The antigenic cross-reactivity of the fusion protein antisera against the parent protein (epsilon toxin) was also evaluated by Western blot analysis. It is clear that the antibodies present in the fusion protein antisera (anti-rLTB.Etx_40–62_ polyclonal antibody) could effectively detect a band of ~ 35 kDa corresponding to the recombinant Etx in Western blot (Fig. 4b). The self-adjuvanting property of the LTB was also established by immunization of the LTB and the rLTB.Etx_40–62_ alone as well as with alum as an adjuvant (Additional file 1: Figure S1A–D). As evident from the figure, significant immune response could be generated when the LTB (Additional file 1: Figure S1A, *p *≤ 0.05 to *p *≤ 0.001 at different dilutions with respect to preimmune serum) and fusion protein (Additional file 1: Figure S1B, *p *≤ 0.005 to *p *≤ 0.001 at different dilutions with respect to preimmune serum) were administered by themselves, confirming the self-adjuvanting activity of the LTB. Immunization of the LTB and the rLTB.Etx_40–62_ with alum as adjuvant (B and D, respectively) augmented the immune response as significantly higher absorbance in ELISA was obtained when compared to the absorbance obtained with the antisera generated against the LTB (Additional file 1: Figure S1C, *p *≤ 0.01 to *p *≤ 0.001 at different dilutions) and the fusion protein alone (Additional file 1: Figure S1D, *p *≤ 0.05 to *p *≤ 0.001). To determine the type of immune response generated by the fusion protein, antibody isotype profiling of the anti-rLTB.Etx_40–62_ antisera was carried out. As evident from Fig. 4c, a significant increase in all isotypes predominantly IgG1 (*p *≤ 0.005), IgG2a (*p *≤ 0.001), IgG2b (*p *≤ 0.001) was observed in comparison to the preimmune sera.

### Neutralization potential of the anti-rLTB.Etx_40–62_ antisera

**Fig. 5:**
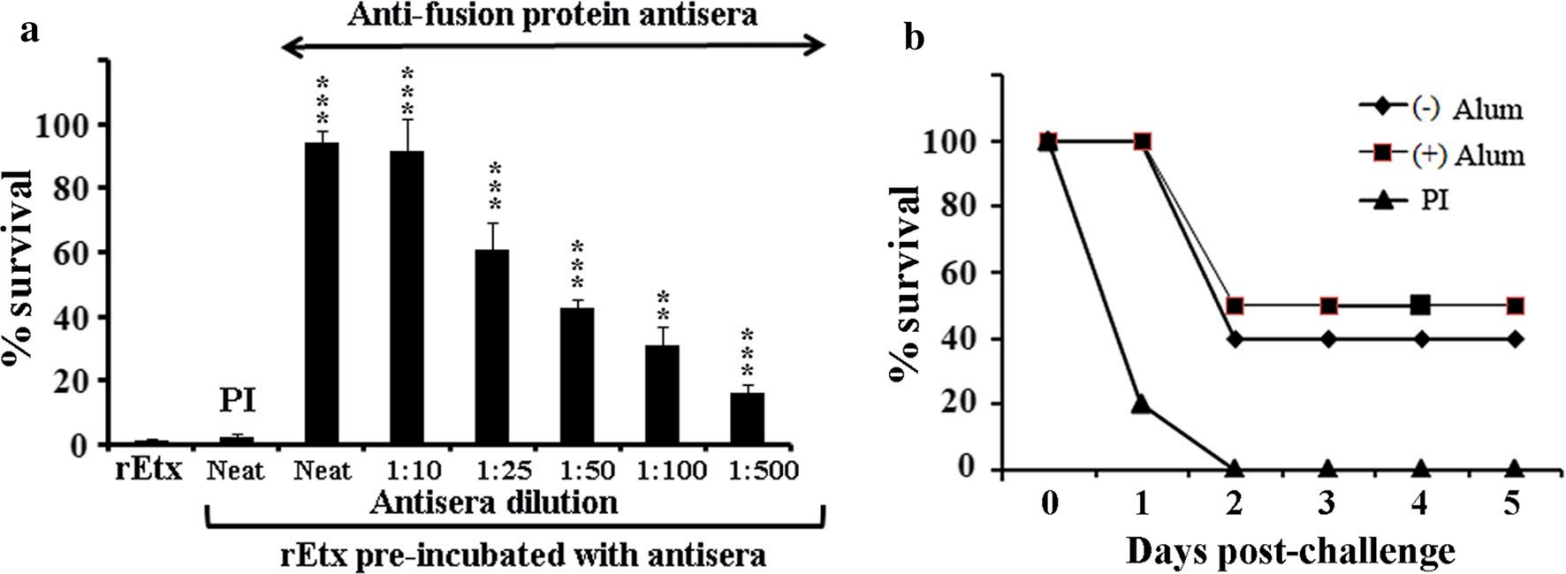
Toxin neutralization capacity of anti-rLTB.Etx_40–62_ antibodies: **a** in vitro neutralization ability of the anti-fusion protein antisera against the epsilon toxin was determined by incubating the rEtx (75 ng/ml) with different dilutions of anti-fusion protein antisera [undiluted (neat), 1:10, 1:25, 1:50, 1:100 and 1:500] prior to its addition to the MDCK cells. The cells were then incubated for 1 h at 37 °C and cell viability was determined by MTT assay. Cells treated with the rEtx pre-incubated with the undiluted pre-immune sera (PI, neat) were included as control. Percent survival of MDCK cells was plotted with respect to untreated control cells. Ordinary one-way ANOVA was performed to determine the statistically significant change (*p* value) in the survival with respect to rEtx-treated cells. **p ≤ 0.005; ***p ≤ 0.001. **b** BALB/c mice (n = 10 per group) were challenged with the 2 × LD50 dose of the rEtx (3 ng/20 g body weight in 5 µl PBS; intraperitoneal) pre-incubated with equal volume of undiluted anti-fusion protein antisera, generated with or without alum or preimmune serum (PI) at 37 °C for 1 h. The mice were monitored for survival for 5 days. Percentage survival was calculated according to the observed mortality

The efficacy of the antisera generated against the rEtx was determined by in vitro neutralization assays using MDCK cells which are susceptible to Etx toxicity (Payne et al. 1994). Notable reduction in the rEtx toxicity was noted when it was pre-incubated with different dilutions of anti-fusion protein antisera prior to its addition to the cells (*p *≤ 0.005 to *p *≤ 0.001). Pre-incubation of the rEtx with the neat anti-fusion protein antisera resulted in significant reduction in the rEtx toxicity (*p *≤ 0.001) with 94.35 ± 4.05% survival of the cells with respect to untreated control cells. The percentage survival of the cells declined in a dilution dependent manner, when the cells were treated with rEtx preincubated with increasing dilution of antisera No significant improvement in the survival was noted when the cells were treated with rEtx pre-incubated with undiluted pre-immune sera. Microscopic analysis of these cells also confirmed the neutralization potential of the Etx toxicity by the fusion protein antisera (Additional file 1: Figure S2). Further, protective efficacy of the antisera was also assessed by administering the mice with 2 × LD50 dose of rEtx pre-incubated with equal volume of antisera (Fig. 5b). As evident from the figure, both the antisera generated with or without adjuvant conferred 50% and 40% protection, respectively, from 2 days post-challenge. The mortality levels of 80% at 1 day post-challenge and of 100% at 2 days post challenge were observed in the group challenged with the rEtx pre-incubated with pre-immune sera.

## Discussion

Use of *V. cholerae* secretory expression system is advantageous to produce heterologous proteins as over expression of recombinant proteins in *E. coli* often results in formation of misfolded inclusion bodies, thus making production of bioactive soluble protein difficult. One of the major concerns in the subunit vaccine production is cost effectiveness. Unlike *E. coli*, *V. cholera*e expression system secrets recombinant proteins in soluble form to the extracellular milieu, simplifying downstream processing and purification, thus bringing down the cost (Ghorpade and Garg 1993; Pugsley 1993; Pillai et al. 1996). We therefore used *V. cholerae* system to express and purify the rLTB.Etx_40–62_ fusion protein. Detection of the purified protein at a position corresponding to its pentamer under non-reducing and non-denaturing conditions, indicates that like the native LTB, the LTB component in the fusion protein could efficiently form a pentamer and that the presence of epitope did not cause any stearic hinderance in pentamerization of LTB. Any conformational change in the native protein due to addition or deletion of amino acids may affect the protein function and stability (Dertzbaugh et al. 1990; Dertzbaugh and Elson 1993). The conformational stability of any antigen has great influence on the intracellular fate of antigens during processing in APCs, thereby greatly affecting the immunogenicity and immune polarization. Structural integrity and conformational stability of proteins is also highly critical for attaining a stable formulation of vaccines (Maddux et al. 2011; Scheiblhofer et al. 2017). In the present study, the secondary structure conformation of the recombinant fusion protein showed no statistically significant difference when compared to the secondary structure conformation of the native LTB. These data suggest negligible change in the secondary structure caused by the fusion of the epitope at the C-terminus of LTB. Presence of an epitope of β-toxin of *C. perfringens* at the C-terminus of LTB did not change its receptor binding activity, indicating no conformation change in the LTB (Bhatia et al. 2014).

Wild type holotoxin, made up of wild type LTB and LTA has been reported to alter CHO-K1 cells morphology (elongation of cells) (Guerrant et al. 1974; Kothary et al. 1995). We have earlier shown that the rLTB.Etx_40–62_ could bind to the GM1 receptor which is crucial for the biological activity of LTB (Kaushik et al. 2013). Analysis of the effect of the holotoxin comprising the rLTB.Etx_40–62_ fusion protein and LTA on CHO-K1 cells further confirmed that the presence of epitope did not affect the biological activity of LTB. The holotoxin comprising the rLTB.Etx_40–62_ was as effective as that comprising the wild type LTB. Both LTB and CTB have been reported to enhance the magnitude of antigen-specific immune responses and have identical activities (Rappuoli et al. 1999; Foss and Murtaugh 2000). However, it has been reported that when used as a fusion partner/adjuvant, LTB resulted in a significantly greater immune response when compared with CTB (Ma 2016).

The high immune response generated against the fusion protein even without any adjuvant signifies that the self-adjuvanting activity of the LTB has been retained in the fusion protein. Previous studies in our lab have demonstrated that immunization of LTB or LTB-fusion proteins without adjuvant results in significant antibody response though the titers are much higher when the proteins are administered with adjuvant (Pillai et al. 1996). We, therefore, preferred to administer the fusion proteins emulsified in alum adjuvant.

Higher antibody titers against LTB are expected as it has been reported to be highly immunogenic and LTB being a larger partner, a significant immune response could be diverted towards this. It has also been shown earlier that the antibody titres generated against the antigenic site when fused with LTB were often lower as compared to the full length LTB, but still have enough to confer protection against the toxin (Mason et al. 1998). The fusion protein expressed as a soluble protein, indicating that the LTB.epitope fusion possibly attained its native conformation which results in appropriate antigen presentation when administered to the animals. Studies have shown that the fusion of HBV surface antigen epitopes (Schodel et al. 1991) with LTB and glycotransferase B epitope (Dertzbaugh and Elson 1993) with CTB retained not only the biological function of CTB as well as antigenicity and immunogenicity of both the fusion partners. LTB has also shown an effective immune response against avian influenza H5N1 virus (Ma 2016; Lei et al. 2015).

Detection of both the rLTB and the rEtx by the anti-rLTB.Etx_40–62_ antisera suggest that the immune system could recognize the immunodominant Etx epitope (40–62 amino acid residues) in the fusion protein and that the epitope spanning 40–62 amino acids was capable of generating an effective Etx-specific immune response. Our results thus suggest that the epitope chosen in the present study contributed significantly towards the antigenicity of the Etx and could generate an antibody response towards the Etx with a highly immunogenic fusion partner LTB. A similar ability of the fusion protein antisera to detect native as well fusion partners has been demonstrated in earlier studies (Bhatia et al. 2014; Sharma and Dixit 2015). Studies have also shown that LTB when used as an adjuvant could generate a strong immune response towards the fused antigens (Pillai et al. 1996; Rock et al. 1996; Marchioro et al. 2014).

For a vaccine to be efficacious and protective, both arms of the immune system i.e. cell mediated and humoral, must be activated. The immunoglobulin isotype (IgG) may differ with the type of immunogen and adjuvant administered and are indicative of the type of immune response generated. An increase in IgG2a and IgG2b isotypes is linked with a Th1 immune response, whereas an increase in the IgG1 and IgG3 isotypes is linked with a Th2 immune response. The antibody isotype profile of the anti-rLTB.Etx_40–62_ antisera indicated generation of a mixed immune response suggesting that immunization with the fusion protein resulted in activation of both the arms of immune response [cell mediated (Th1) as well as humoral (Th2)], which is desirable for an effective vaccine. A similar immune response has been observed with a multi-epitope vaccine against *Brucella* *melitensis* (Yin et al. 2016) and a subunit vaccine against leishmania (Mazumdar et al. 2004), and also when LTB was used as an adjuvant and administered orally (Marchioro et al. 2014; Nakagawa et al. 1996).

Both the in vitro and in vivo challenge studies using rEtx showed that the anti-rLTB.Etx_40–62_ antisera was able to neutralize the toxin. These data thus demonstrate that the epitope present in the fusion protein was capable of generating Etx neutralizing antibodies. Our results are in agreement with earlier reports where in a vaccine containing a specific epitope could produce the potent broadly neutralizing antibodies against the antigen and could effectively prevent the *Streptococcus agalactiae*, *Streptococcus aureus* and *Mycobacterium tuberculosis* H37Rv infection (Xu et al. 2011; Christy et al. 2012). Greater effectiveness of epitope/subunit-based vaccine in comparison to the whole antigen have been reported for both viral and bacterial infection (Zhao et al. 2015; Goldenberg et al. 2016). Although use of formaldehyde treated culture filtrates are currently used for vaccination against *C. perfringens*, the neutralizing effect can vary in protective efficacy (Lobato et al. 2010). Use of defined recombinant protein production under controlled conditions overcomes this problem of inconsistency.

Thus, in the present study we have demonstrated that use of genetic fusion of an immunodominant epitope of Etx with LTB generated an effective immune response capable of negating epsilon toxin toxicity both in vitro and in vivo, and demonstrated the potential of Etx epitope-LTB fusion proteins as a candidate vaccine against *C. perfringens* epsilon toxin.

## Additional file

**Additional file 1:Fig. S1.** Additional Antibody titer determination.** Fig. S2**. Microscopic analysis of MDCK cells treated with the rEtx pre-inncubated with anti-rLTB.Etx40-62 antisera.

## Data Availability

Data sharing not applicable to this article as no datasets were generated or analysed during the current study.
