# Supplementary material for: Immunization with recombinant fusion of LTB and linear epitope (40–62) of epsilon toxin elicits protective immune response against the epsilon toxin of Clostridium perfringens type D
Source: AMB Express. 2019 Jul 12;9:105. doi: 10.1186/s13568-019-0824-3 (PMC6626085; doi:10.1186/s13568-019-0824-3)
Supplement: Supplementary file 1 — Additional file 1:Fig. S1. Additional Antibody titer determination. Fig. S2. Microscopic analysis of MDCK cells treated with the rEtx pre-inncubated with anti-rLTB.Etx40-62 antisera. [file 13568_2019_824_MOESM1_ESM.pdf]

AMB Express

Additional File 1

Immunization with recombinant fusion of LTB and linear epitope (40-62) of epsilon toxin elicits protective immune response against the epsilon toxin of *Clostridium perfringens* type D.

Himani Kaushik[1], Sachin K. Deshmukh[1], Amit Kumar Solanki[1], Bharati Bhatia[1], Archana Tiwari[2] and Lalit C. Garg[1]

[1]Gene Regulation Laboratory, National Institute of Immunology, Aruna Asaf Ali Marg, New Delhi – 110067, INDIA

[2]School of Biotechnology, Rajiv Gandhi Prodyogiki Vishwavidyalaya, Airport Bypass Road, Gandhi Nagar, Bhopal, - 462035, INDIA

Corresponding author

Dr. Lalit C. Garg,

Gene Regulation Laboratory, National Institute of Immunology,

Aruna Asaf Ali Marg, New Delhi – 110067, India.

Tel.: +91 11 2670 3652; fax: +91 11 2674 2125.

Email: lalitcgarg@gmail.com; lalit@nii.ac.in

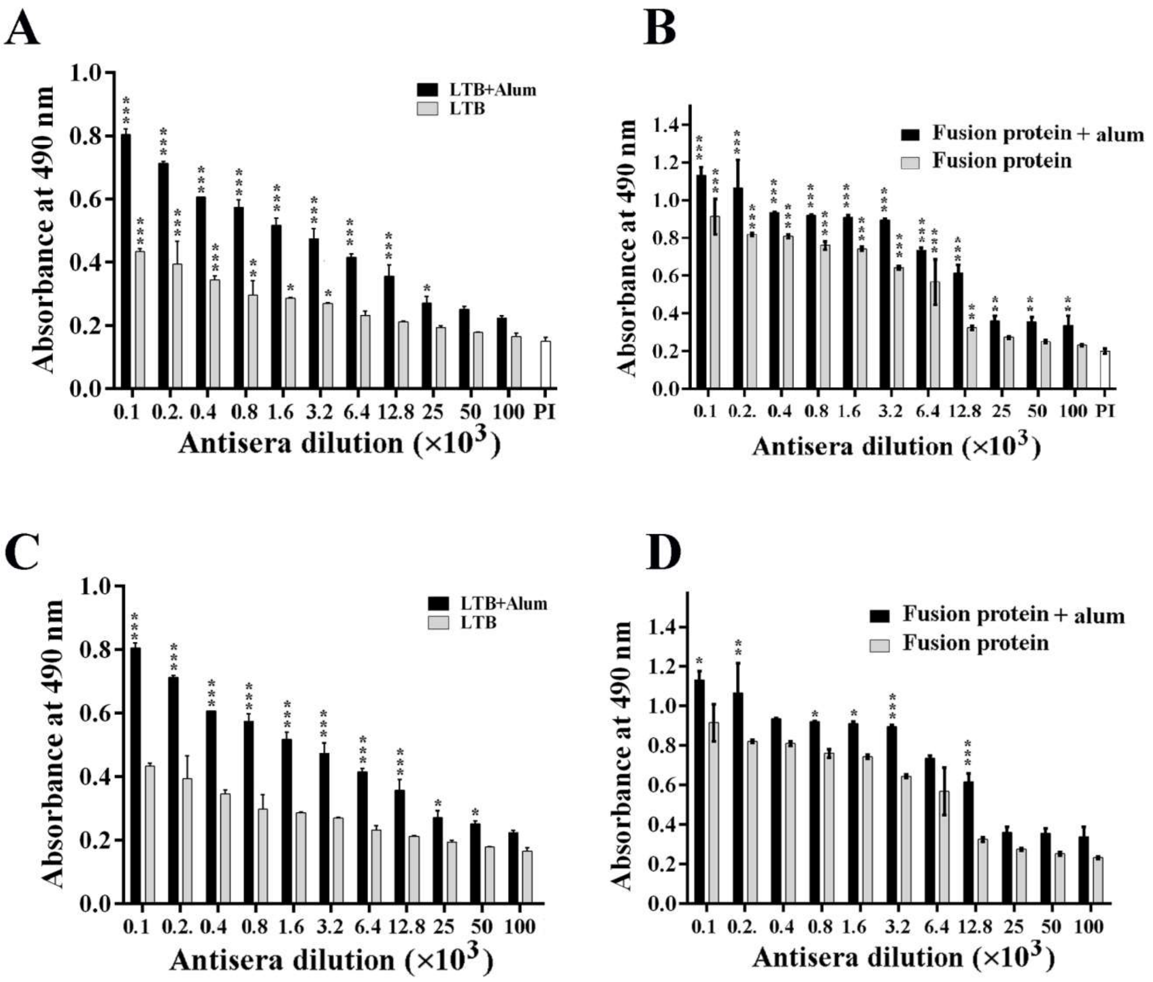

Fig. S1. Antibody titer determination: ELISA to determine antibody titers in the antisera from mice immunized with (A) LTB alone and LTB with alum as adjuvant and (B) Fusion protein rLTB.Etx$_{40-62}$ alone and Fusion protein rLTB.Etx$_{40-62}$ with alum as adjuvant. As evident from the figure, significant immune response could be generated when the LTB and fusion protein were administered by themselves, signifying the self-adjuvanting activity of the LTB. Immunization of these proteins with alum as adjuvant resulted in an increase in the immune response (Figures S1C and S1D).

Figures S1A and S1B show the statistical difference (*p* value) calculated using ordinary two-way ANOVA with respect to pre-immune (PI) serum. Figures S1C and S1D show the statistical difference (*p* value) between the antisera generated with alum as compared to that generated without alum. *, $p \leq 0.05$; **, $p \leq 0.005$; ***, $p \leq 0.001$.

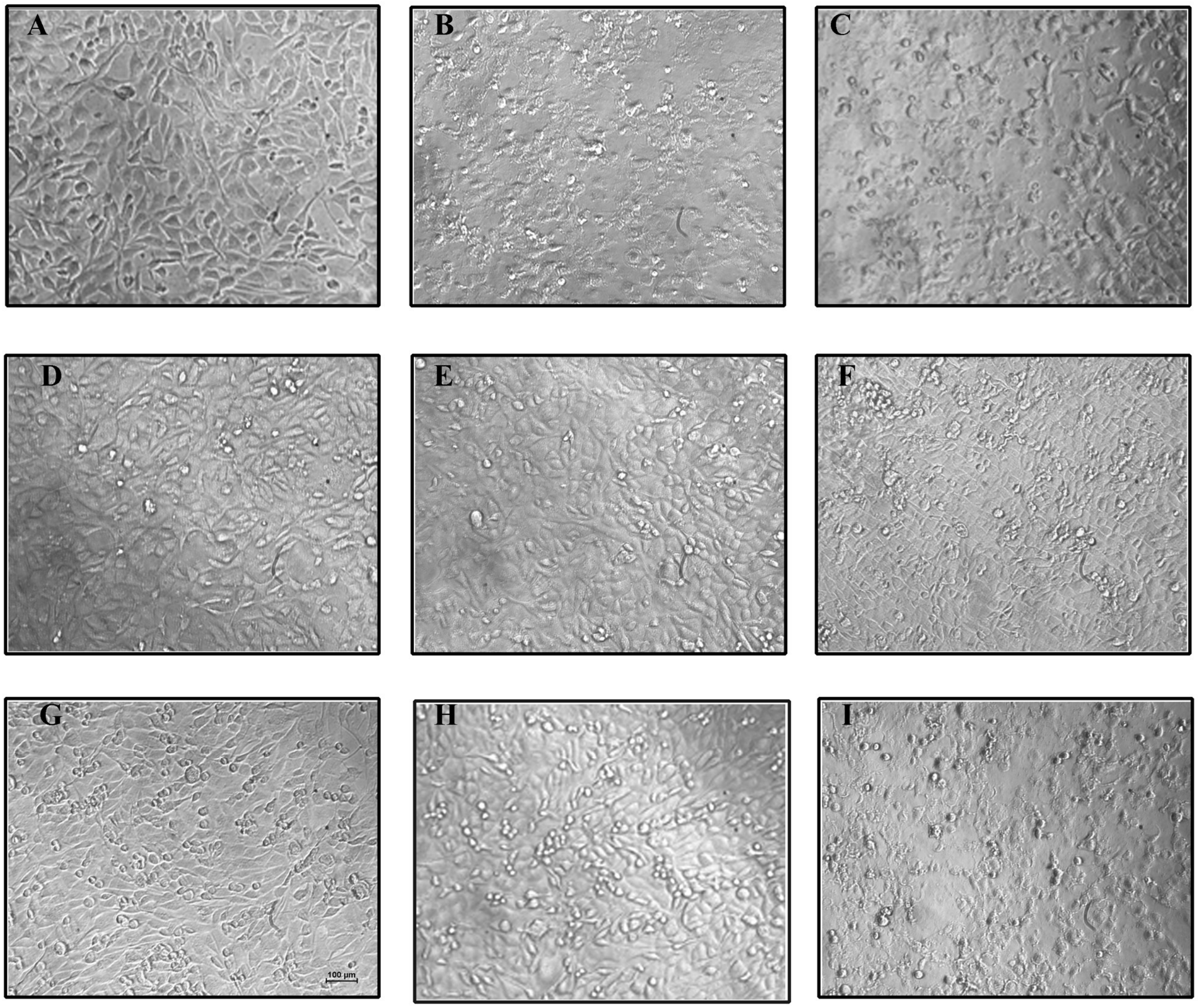

Fig. S2. Microscopic analysis of MDCK cells treated with the rEtx pre-inncubated with anti-rLTB.Etx40-62 antisera: The rEtx (7.5 ng/5 µl) was pre-incubated with equal volume of different dilutions of the anti-rLTB.Etx_{40-62} antisera collected after the 2nd booster for 1 h at 37°C prior to the addition to MDCK cells. The MDCK cells (2×10^{4}/100 µl/well) were treated with the rEtx alone or antisera-toxin mixture at for 2 h at 37°C and 5% CO_2 in a humidified incubator. **(A)** Control cells in the culture medium. **(B)** Cells treated with the rEtx. **(C)** Cells treated with rEtx pre-incubated with equal volume of preimmune sera. **(D-I)** Cells treated with the rEtx preincubated with different dilutions of anti-rLTB.Etx_{40-62} antisera (D, neat; E,1:10; F, 1:25; G,1:50; H,1:100 and I, 1:500). Images are acquired at 20×maginification using a light microscope.
